# Notes

**Published:** 1900-04

**Authors:** 


### NOTES.

Bridgeton, N. J., has become seriously alarmed at the prevalence of tuberculosis and her high death-rate, and will establish at once a milk-inspection system.

The Iowa Legislature has a bill before that body providing for a lien on foals for the stallion fee.

The purchase of many of our fast trotters and pacers, our young stallions and brood mares of note, continues to a greater degree, an evidence of the progress of the United States in developing the horse of speed.

The *Rider and Driver* now carries a column for its veterinary subscribers, placing the name, address, and telephone number in this directory.

A Missouri law requiring a tuberculin test of all cattle entering the State for breeding purposes went into effect January 1, 1900.

The advocates of the Holstein-Friesian breed of cattle are daily growing more confident of the value of these animals for dairy purposes, with many advantages to their credit over the generally selected Channel Island cattle. The reckless breeding of the Jerseys, decreasing them in vigor and size, was a result somewhat disastrous to this breed,whereas those having the Holstein-Friesian at heart have continued to blend their higher grade ones and maintained size and weight, and, while maintaining their value as prolific milkers, they have not lost sight of increasing the quality of the milk and making them better butter producers. When no longer valuable for the dairy they can be profitably turned to beef, a point not attained by the Jersey.

